# Influence of Polyurea Composite Coating on Selected Mechanical Properties of AISI 304 Steel

**DOI:** 10.3390/ma12193137

**Published:** 2019-09-26

**Authors:** Monika Duda, Joanna Pach, Grzegorz Lesiuk

**Affiliations:** 1 Department of Mechanics, Materials Science and Engineering, Wrocław University of Science and Technology, Smoluchowskiego 25, 50-370 Wrocław, Poland; monika.duda@pwr.edu.pl; 2 Department of Foundry, Polymers and Automation, Wrocław University of Science and Technology, Smoluchowskiego 25, 50-370 Wrocław, Poland; joanna.pach@pwr.edu.pl

**Keywords:** AISI 304, polyurea, composite coating, impact resistance, adhesion, delamination, fatigue

## Abstract

This paper contains experimental results of mechanical testing of the AISI 304 steel with composite coatings. The main goal was to investigate the impact of the applied polyurea composite coating on selected mechanical properties: Adhesion, impact resistance, static behavior, and, finally, fatigue lifetime of notched specimens. In the paper the following configurations of coatings were tested: EP (epoxy resin), EP_GF (epoxy resin + glass fabric), EP_GF_HF (epoxy resin + glass fabric hemp fiber), EP_PUA (epoxy resin + polyurea) resin, EP_GF_PUA (epoxy resin + glass fabric + polyurea) resin, and EP_GF_HF_PUA (epoxy resin + glass fabric + hemp fiber + polyurea) resin. The highest value of force required to break adhesive bonds was observed for the EP_PUA coating, the smallest for the single EP coating. A tendency of polyurea to increase the adhesion of the coating to the base was noticed. The largest area of delamination during the impact test was observed for the EP_GF_HF coating and the smallest for the EP-coated sample. In all tested samples, observed delamination damage during the pull-off test was located between the coating and the metallic base of the sample.

## 1. Introduction

Polymer coatings are the subject of a lot of research in a number of publications [1,2,3,4,5]. They are applied as anti-corrosive agents [6,7,8,9] as well as an anti-wear agents preventing abrasion, tearing, and scratches [10,11,12,13] due to their specific mechanical properties. In those roles, polyurea, polyurethane, and polyurethane–polyurea resins are mainly used.

Polyurea coatings are increasingly popular in recent years [14,15,16]. They can be applied on metallic, wooden, and concrete surfaces, or even other plastics. These coatings allow for desired decorative properties to be obtained as well as specific mechanical properties. Polymers are used as cover layers for armed vehicles, ballistic shields [17], loading area of vehicles, and as a waterproof layer on concrete surfaces [18,19]. Additionally, they can absorb vibrations and sound waves. In order to strengthen the layers’ properties, they were modified with glass fabric and hemp fiber.

The issue regarding the application of coatings is its poor adhesion to a metal surface. Due to this, in industrial conditions the surface is pre-processed by sandblasting and/or by application of an intermediate layer—primer—based on epoxy resin.

According to the practice of the polyurea coating application process, in the investigation presented in this paper the epoxy resin was used as an intermediate layer. The primer was modified in order to improve the impact resistance and vibration absorption by glass fabric and hemp fiber. In order to determine the influence of each constituent of the layer, there were also prepared samples with and without the polyuria layer. In order to eliminate the influence of mechanical treatment on the results of the experiment, the metallic base material was cleansed with acetone; the sand blasting process was not applied.

The coating was modified with natural fibers due to their low density and good ability to suppress acoustic waves. This application is often present in the automotive industry, where natural fibers are used as a filler in composite elements of vehicle interiors [20]. An important technological limitation in the natural fiber-reinforced composites industry is the temperature, which should not be bigger than 230 °C. Exceeding that temperature would cause the degradation of the fiber. Nevertheless, it is not an issue while using chemo-hardening resins.

The aim of the study was to determine the adhesion force of the polymeric coatings to the steel base, to compare the impact resistance of multilayer coatings based on the damage analysis caused by the impact of the energy of 17 J, to determine the coating resistance to cracking and peeling from the base as well as to investigate the influence of the coating on static mechanical properties and the fatigue lifetime of the sample, which is expected to improve. In general, the most widely used strategy of fatigue lifetime improvement is strengthening metallic structures using CFRP (carbon fiber reinforced polymer) patches [21,22,23]. The main reason for this is the redistribution of forces in metallic and composite structures. In this paper, the beneficial effect of polyurea composite on the fatigue performance of AISI 304 steel will be also demonstrated.

## 2. Materials and Methods

As a base material, austenitic steel AISI 304 in the form of 0.5 mm thick metal sheet was used. Chemical composition and static tensile results [24] of AISI 304 ((0.04%C, 1.1%Mn, 0.41%Si, 0.0437%P, 0.0044%S, 18.16%Cr, 8%Ni, 0.0335%Mo, 0.1%V, 0.32%Cu) steel are included in Table 1.

**Figure 1:**
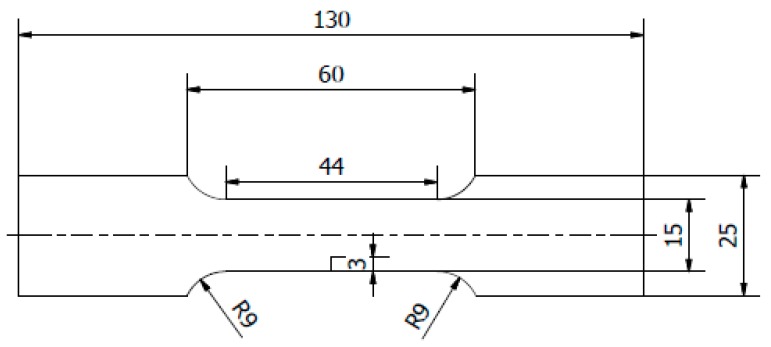
The geometry of a notched sample.

The base was degreased with acetone. For the adhesion and impact tests 100 × 100 mm samples were prepared; for static tensile and fatigue tests oar-shaped samples were prepared (Figure 1).

The type of applied coatings and layers configurations are presented in Table 2.

**Figure 2:**
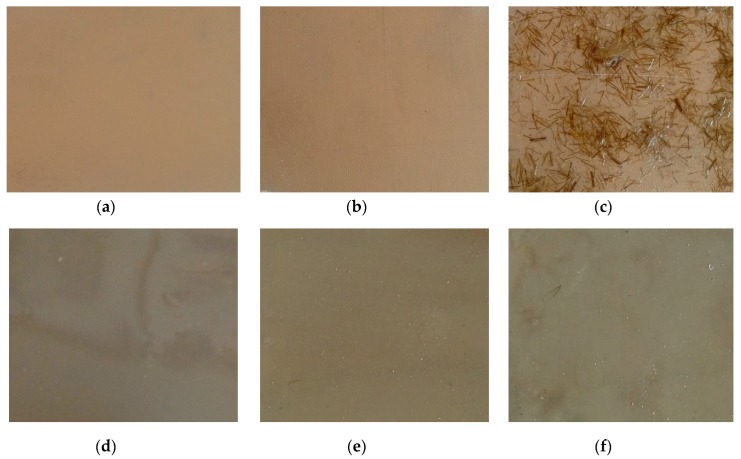
Images of the obtained surfaces: (**a**) EP; (**b**) EP_GF; (**c**) EP_GF_HF; (**d**) EP_PUA; (**e**) EP_GF_PUA; (**f**) EP_GF_HF_PUA.

The coatings were applied manually. All samples were coated with epoxy resin LH 289 and characterized by low viscosity (Havel composites); more information about the resin are presented in Table 3. The first layer of the coating was modified by reinforcing it with glass fiber Areoglas 163 g/m^2^ (Table 4) and/or with cut hemp fibers of 40 mm length. Part of the samples was covered with two-component polyurea coating Almacoat Floor Sl (Table 5). Obtained surfaces of the samples are presented in Figure 2.

## 3. Results

### 3.1. Adhesion of Coatings

**Figure 3:**
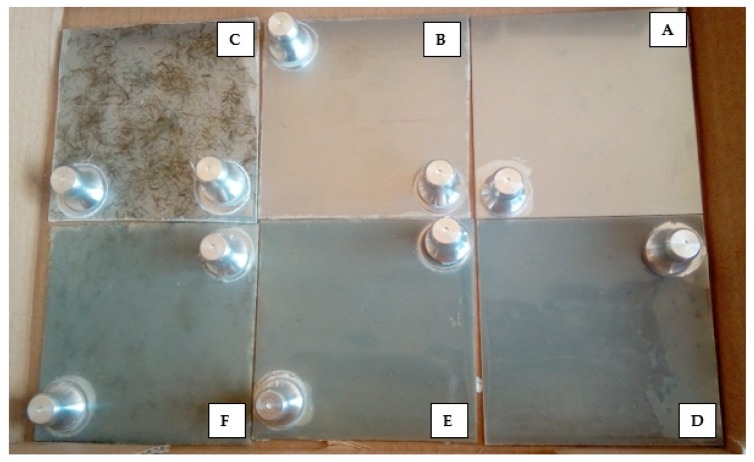
Samples prepared for adhesion testing, A—EP; B—EP_GF; C—EP_GF_HF; D—EP_PUA; E—EP_GF_PUA; F—EP_GF_HF_PUA.

Measurements of the adhesion of the coatings were carried out using a pull-off method, according to the standard PN-EN ISO 4624:2016-05 [25] using the PosiTest AT-A device (DeFelsko Corporation, Ogdensburg, NY, USA). During the test, the pull-off force of the stamp from the polymer coating based on the steel base was estimated. Prior to the test, measurement stamps were applied to the coating. After curing the glue, the circular notch around the stamp was cut and, subsequently, the pull-off test was carried out. There were five measurements done per each type of coating. In Figure 3 images of samples with glued measurement stamps are presented. The pull-off test classification according to the standard PN–EN ISO 4624:2016 [25] is presented in Table 6.

**Figure 4:**
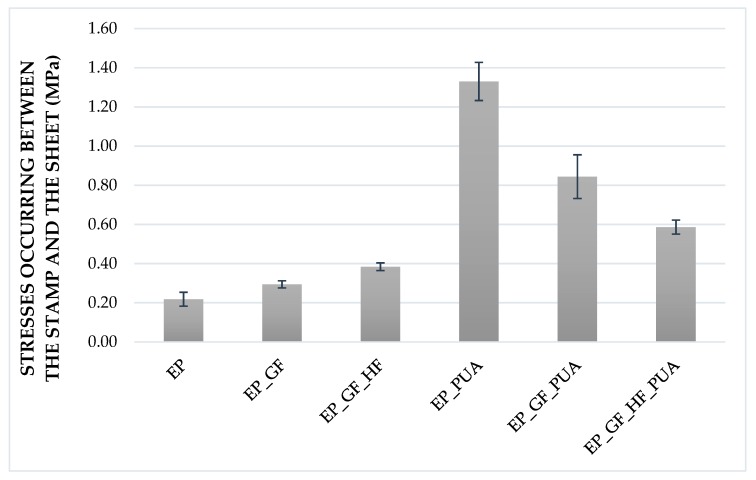
Pull-off force values obtained in the PosiTest test for all types of coating.

The results of adhesion measurements are presented in Table 7. In all cases adhesive separation between the base and the first layer of the coating was obtained. In Figure 4, the results of the pull-off force measurement obtained during the PosiTest test are shown. Due to the different materials (including fibers) used for the coating, the total thicknesses of the layers were different. However, this did not change the reinforcement and redistribution of stresses, as shown by the results of the static tests. During the application of the layers, it was ensured that the thickness of the layers was equally distributed. Quality control with optical scanners revealed differences in thickness not greater than 8% of the applied layer.

### 3.2. Coatings Impact Resistance

**Figure 5:**
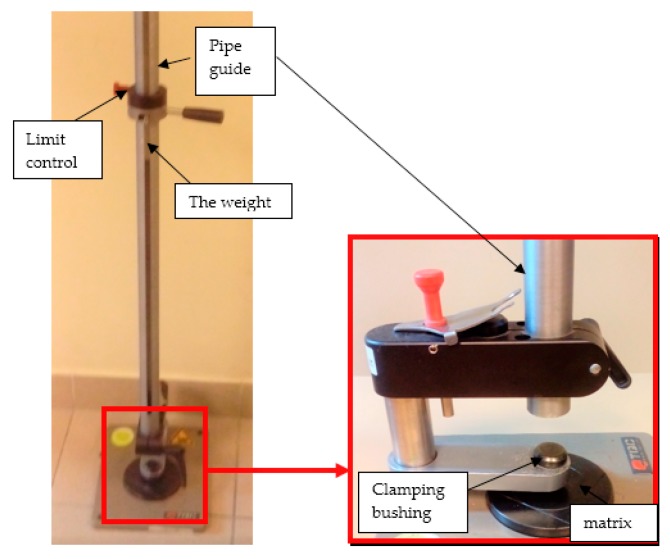
The test stands for evaluation of samples’ impact resistance—general view and the magnification of the base of the test stand.

Coatings resistance to cracking or peeling from the base was evaluated according to the standard PN-EN ISO 6272-1:2011 [26] using the impact resistance testing device—TQC (TQC Sheen B.V., Capelle aan den IJssel, Netherlands), presented in Figure 5. The test consists of determining the minimum height of fall for 20 mm diameter mass, under normalized conditions, in order to damage investigated coatings. The research according to this procedure was conducted also in papers [27,28].

**Figure 6:**
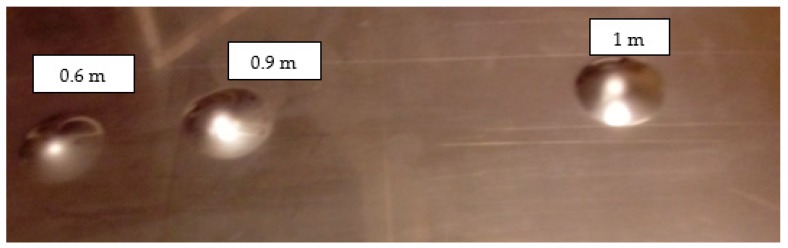
Tested metal sheet deformation after the impact of 1 kg weight from different heights: 0.6, 0.9, and 1.0 m.

**Figure 7:**
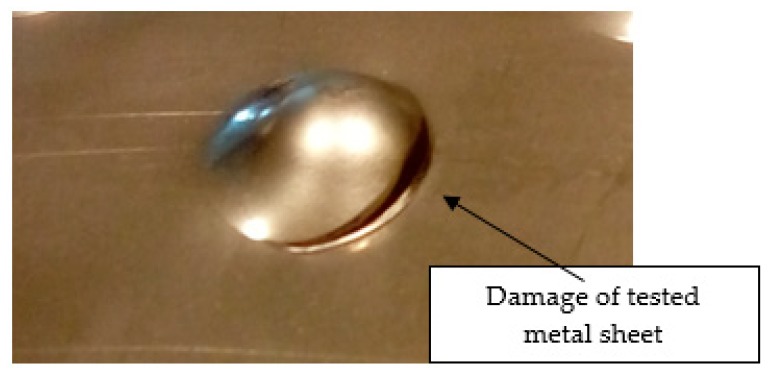
Tested metal sheet damage after the impact of 2 kg weight from 0.9 m height.

Initial tests were conducted on the metallic base without any coating with a load of 1 kg. The metal sheet was hit from different heights and there was no observed rapture of the material (Figure 6). Due to the lack of visible material damage after dropping the weight of 1 kg from maximal height of 1 m, the weight was changed to 2 kg. Material damage was observed for the drop of 2 kg weight from 0.9 m height. The obtained sample was used as a reference sample for further tests on coated samples (Figure 7).

The impact resistance test was conducted on previously-prepared coated samples, using the weight of 2 kg gravity dropped from 0.9 m height. Observed results of the test are presented in Table 8 and Table 9.

### 3.3. Static Tensile Test of Notched Specimens

**Figure 8:**
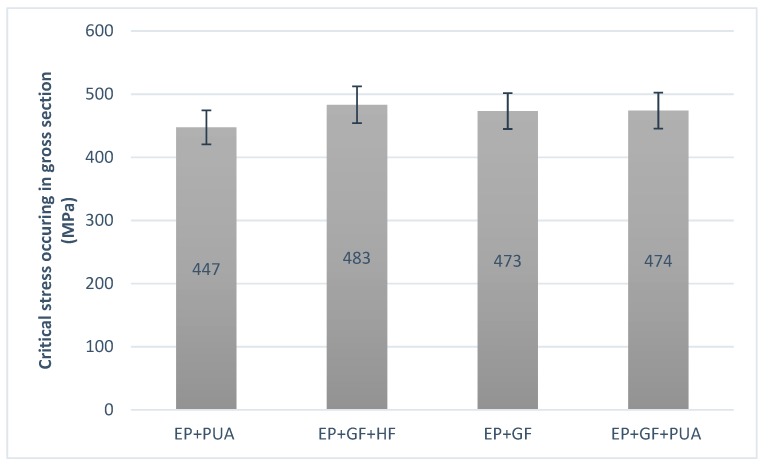
Comparison of the failure load during static tensile test.

The static tensile test was conducted on MTS 810 Material Testing Machine (MTS Systems Corporation, Eden Prairie, MN, USA). The test was conducted for samples with EP_GF, EP_PUA, EP_GF_PUA, and EP_GF_HF coatings. The results are the mean of tests on five samples per coating and presented in Figure 8. All results correspond well with the previously obtained [29] experimental data for the same specimen configuration (without coating) made from AISI 304 steel. The critical gross-section tensile stress for the notched (k_t_ = 5.88) AISI 304 steel specimen was estimated at the level 505 MPa. The results for the sample with EP coating were no different from the value for non-coated steel. 

### 3.4. Fatigue Testing

**Figure 9:**
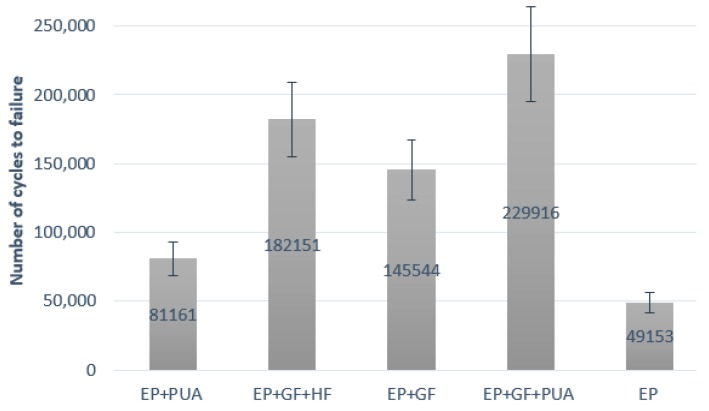
Number of cycles to failure (mean of five) for samples with different coatings.

The fatigue test was conducted on the uniaxial MTS 810 Material Testing Machine equipped with a 5 kN load cell under a stress-controlled mode for one selected load level. During the test stress, ratio (R = 0.05) and frequency (f = 20 Hz) were kept constant. All specimens were loaded using sinusoidal waveform with the maximum load level F_MAX_ = 1400 N and minimum load level F_MIN_ = 70 N. In order to achieve proper surface and geometry of the notch, as well as to avoid delamination of the coating, the notch was cut out using the diamond string method. Obtained results are the mean of five samples and presented in Figure 9.

**Figure 10:**
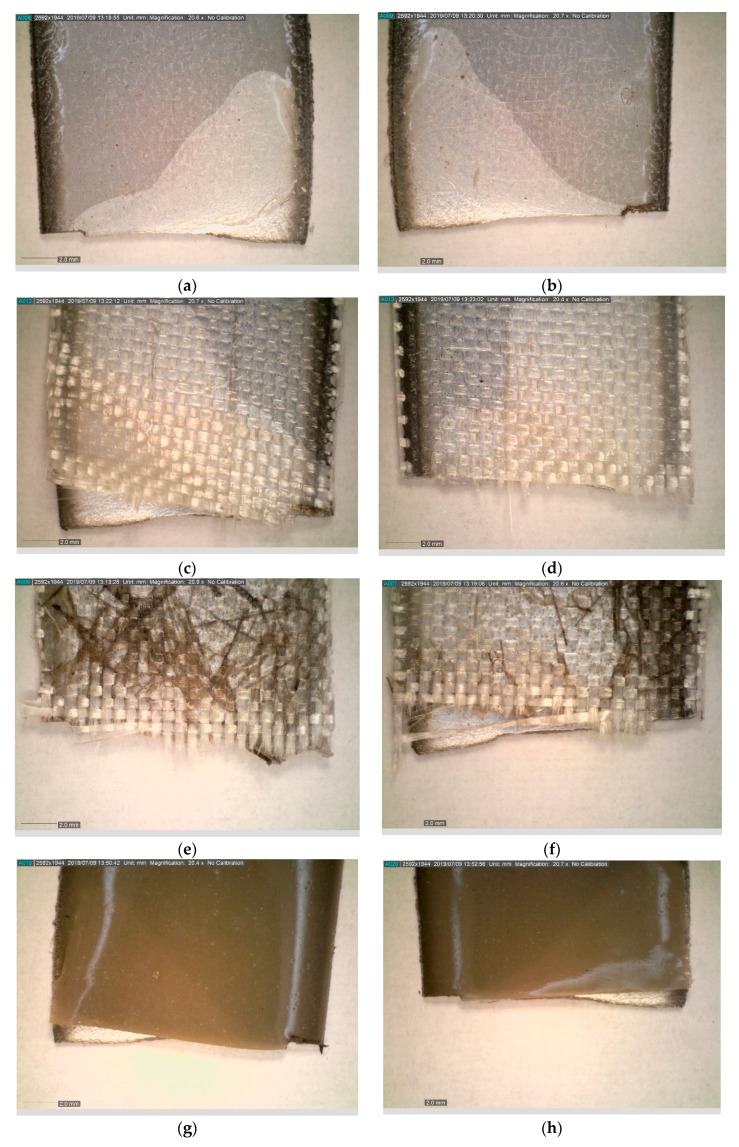

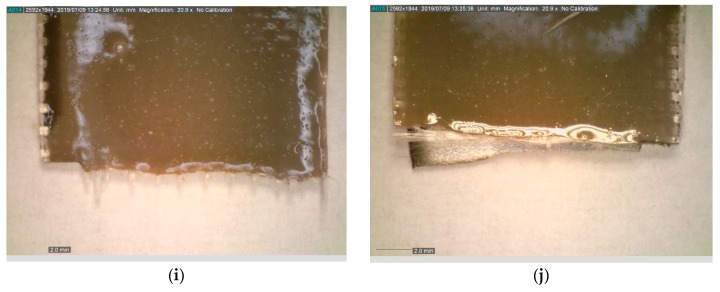
Images of the fracture area after fatigue test for samples: (**a**,**b**) EP; (**c**,**d**) EP_GF; (**e**,**f**) EP_GF_HF; (**g**,**h**) EP_PUA; (**i**,**j**) EP_GF_PUA.

The macroscopic images of broken specimens are presented in Figure 10.

Coatings with glass fiber as a constituent had a visible asymmetric fracture surface. In case of all samples, there was visible delamination of the coating from the metallic base. The area of delamination was different for each sample, including samples with the same coating. Nevertheless, the delamination process was observed to start behind the notch area, or was even not observed in the notch area at all. This indicates that the notching method was selected and conducted properly. There was no visible delamination between the layers of the coating. The observed break of adhesion forces was, in the case of all samples, between the metallic base and the coating.

## 4. Conclusions

Based on the performed experimental campaign, the following conclusions can be drawn:

(1) It was observed that the highest value of force required to break adhesive bonds was achieved for the EP_PUA coating, the smallest for the single EP coating.

(2) The largest area of the delamination during the impact test was observed for the EP_GF_HF-coated sample and the smallest for the EP-coated sample.

(3) The static tensile test did not show a significant difference in the influence of the coating on the tensile strength of the material.

(4) Fatigue tests results showed that the difference in the number of cycles to failure depends on the type of coating used. For coatings with polyurea and glass fiber as constituents, the increase of fatigue lifetime was significant.

(5) The macroscopic analysis of the fracture area of damaged samples confirms that the method of notch preparation was correct and had no influence on the behavior of individual samples during the fatigue test.

Due to the possibility of manual application of the coating, if further research on the fatigue lifetime and fatigue crack growth confirm the preliminary results presented in this paper, the coating might be used as an “on-site” fatigue lifetime enhancer and fatigue crack growth retardation tool on the existing structures.

## Figures and Tables

**Table 1:** Static mechanical properties of the analyzed steel AISI304, based on [24].

Material	Ultimate Tensile Strength UTS (MPa)	Yield Strength R_pl_/R_0.2_ (MPa)	Young Modulus E (GPa)	Poisson Ratio ν (-)	Vickers Hardness HV (-)	Elongation at Break A_5_ (%)
AISI 304 steel	612	312	187	0.29	252	57

**Table 2:** Types of applied coatings.

Sample Designation	Composition and Configuration of the Coating
EP	epoxy resin
EP_GF	epoxy resin + glass fabric
EP_GF_HF	epoxy resin + glass fabric hemp fiber
EP_PUA	epoxy resin + polyurea resin
EP_GF_PUA	epoxy resin + glass fabric + polyurea resin
EP_GF_HF_PUA	epoxy resin + glass fabric + hemp fiber + polyurea resin

**Table 3:** Properties of used epoxy resin.

**Molecular weight (g/mol)**	180–193
**Color**	Max.3
**Epoxide index, mol/1000**	0.51–0.56
**Ignition temperature, °C**	above 150
**Viscosity (mPa, 25 °C)**	500–900
**Density (g/cm^3^)**	1.12–1.16

**Table 4:** Properties of used glass fiber.

**Surface mass**		160 ± 10 (g/m^2^)
**Plait**		Plain weave
**Edges**		cut
**Matrix density**		120 ± 1
**Storage**	temperature	Up to 25 °C
	humidity	Up to 68%

**Table 5:** Properties of a used polyurea resin.

Viscosity (25 °C)	ISO-7000 mPas, Polyol-500 mPa	EN ISO 2555 (Brookfield)
**Volatiles**	0%	-
**Density (25 °C)**	ISO-1.10 g/cm^3^, Polyol-1.05 g/cm^3^,	EN ISO 1675
**Life time after mixing (20 °C)**	9 min	-
**Treatment time after effusion (20 °C)**	20 min	-
**Application temperature**	+10 °C to 30 °C	-
**Mixing proportions ISO:Polyol**	100:13 (weight)	-
**Recommended thickness**	2 mm	-
**Tensile strength**	13 MPa	EN ISO 527
**Elongation**	650%	EN ISO 527
**Adhesion to the base (steal)**	>5 MPa	EN ISO 4624
**Adhesion to the base (concrete)**	Rapture in concrete	EN 1542
**Shore’s hardness**	80A	EN ISO 868
**Water absorption (7 days)**	Up to 3.5%	-

**Table 6:** Pull-off test classification according to the norm PN–EN ISO 4624:2016 [25].

Designation	Description
A	Cohesive separation in the base
A/B	Adhesive separation between the base and the first layer
B	Cohesive separation in the first layer
B/C	Adhesive separation between the first and the second layer
N	Cohesive separation in the n-th layer of the system
n/m	Adhesive separation between the n-th and the m-th layer of the system
-/Y	Adhesive separation between the last layer and the adhesive
Y	Cohesive separation in the adhesive
Y/z	Adhesive separation between the stamp and the adhesive

**Table 7:** Results of the adhesion pull-off tests.

Sample Designation	Macroscopic Image	Stresses Occurring between the Stamp and the Sheet (MPa)	Type of Separation
EP	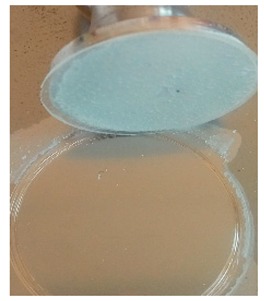	0.22	A/B
EP_GF	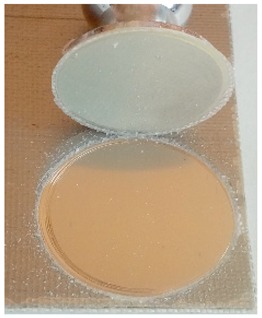	0.29	A/B
EP_GF_HF	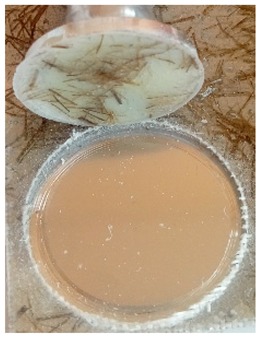	0.38	A/B
EP_PUA	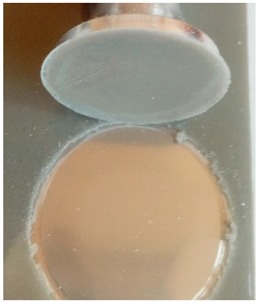	1.33	A/B
EP_GF_PUA	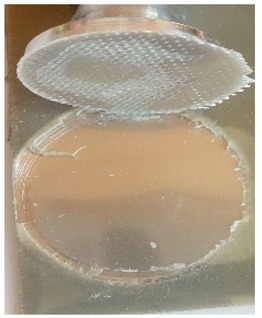	0.84	A/B
EP_GF_HF_PUA	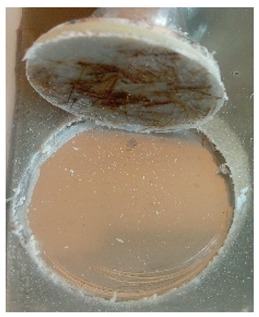	0.59	A/B

**Table 8:** Damage observed after the impact resistance test.

**EP Sample**	
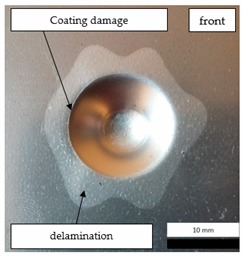	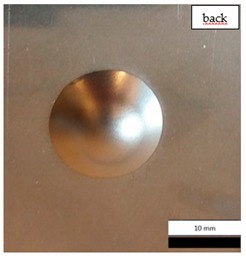
**EP_GF Sample**	
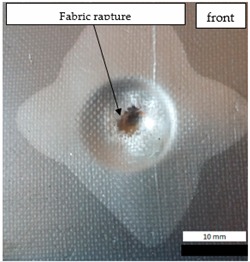	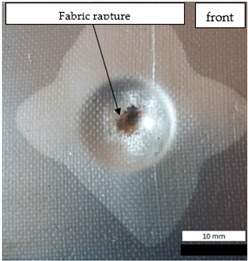
**EP_GF_HF Sample**	
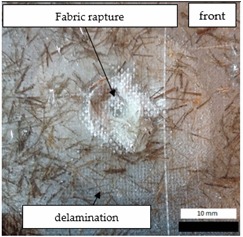	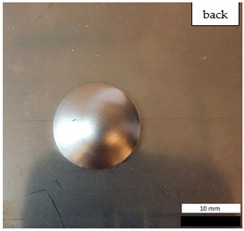
**EP_PUA Sample**	
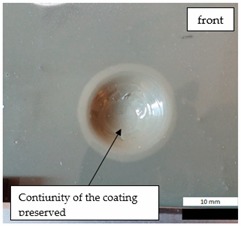	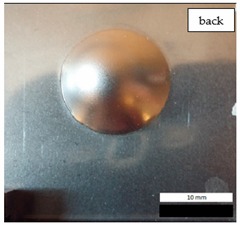
**EP_GF_PUA Sample**	
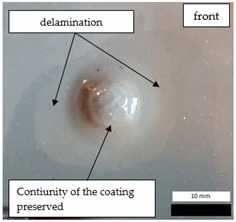	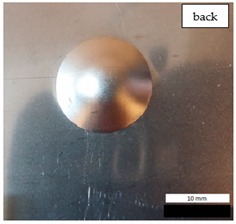
**EP_GF_HF_PUA Sample**	
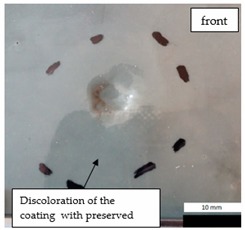	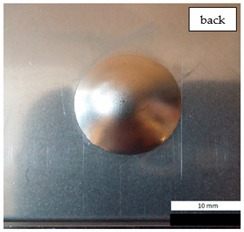

**Table 9:** Coating damage after the impact resistance test.

Sample Designation	Delamination Surface Area [mm^2^]	Remarks
EP	70	Coating delamination, breakage at the edge of the deformation
EP_GF	152	Coating delamination and glass fiber rapture
EP_GF_HF	196	Coating delamination and glass fiber rapture
EP_PUA	53	Lack of damage and delamination of the coating, continuity preserved
EP_GF_PUA	96	Coating delamination, lack of damage, continuity preserved
EP_GF_HF_PUA	166	Coating delamination, lack of damage, continuity preserved
